# Enhanced motor noise in an autism subtype with poor motor skills

**DOI:** 10.1186/s13229-024-00618-0

**Published:** 2024-09-03

**Authors:** Veronica Mandelli, Isotta Landi, Silvia Busti Ceccarelli, Massimo Molteni, Maria Nobile, Alessandro D’Ausilio, Luciano Fadiga, Alessandro Crippa, Michael V. Lombardo

**Affiliations:** 1 Center for Mind/Brain Sciences, University of Trento, Rovereto, Italy; 2 Laboratory for Autism and Neurodevelopmental Disorders, Center for Neuroscience and Cognitive Systems, Istituto Italiano di Tecnologia, Rovereto, Italy; 3 Scientific Institute, IRCCS Eugenio Medea, Bosisio Parini, Italy; 4 Center for Translational Neurophysiology of Speech and Communication, Istituto Italiano di Tecnologia, Ferrara, Italy; 5 Department of Neuroscience and Rehabilitation, University of Ferrara, Ferrara, Italy

**Keywords:** Motor, Stratification, Kinematics, Subtypes, Clustering

## Abstract

**Background:**

Motor difficulties are common in many, but not all, autistic individuals. These difficulties can co-occur with other problems, such as delays in language, intellectual, and adaptive functioning. Biological mechanisms underpinning such difficulties are less well understood. Poor motor skills tend to be more common in individuals carrying highly penetrant rare genetic mutations. Such mechanisms may have downstream consequences of altering neurophysiological excitation-inhibition balance and lead to enhanced behavioral motor noise.

**Methods:**

This study combined publicly available and in-house datasets of autistic (n = 156), typically-developing (TD, n = 149), and developmental coordination disorder (DCD, n = 23) children (age 3–16 years). Autism motor subtypes were identified based on patterns of motor abilities measured from the Movement Assessment Battery for Children 2nd edition. Stability-based relative clustering validation was used to identify autism motor subtypes and evaluate generalization accuracy in held-out data. Autism motor subtypes were tested for differences in motor noise, operationalized as the degree of dissimilarity between repeated motor kinematic trajectories recorded during a simple reach-to-drop task.

**Results:**

Relatively ‘high’ (n = 87) versus ‘low’ (n = 69) autism motor subtypes could be detected and which generalize with 89% accuracy in held-out data. The relatively ‘low’ subtype was lower in general intellectual ability and older at age of independent walking, but did not differ in age at first words or autistic traits or symptomatology. Motor noise was considerably higher in the ‘low’ subtype compared to ‘high’ (*Cohen’s d* = 0.77) or TD children (*Cohen’s d* = 0.85), but similar between autism ‘high’ and TD children (*Cohen’s d* = 0.08). Enhanced motor noise in the ‘low’ subtype was also most pronounced during the feedforward phase of reaching actions.

**Limitations:**

The sample size of this work is limited. Future work in larger samples along with independent replication is important. Motor noise was measured only on one specific motor task. Thus, a more comprehensive assessment of motor noise on many other motor tasks is needed.

**Conclusions:**

Autism can be split into at least two discrete motor subtypes that are characterized by differing levels of motor noise. This suggests that autism motor subtypes may be underpinned by different biological mechanisms.

**Supplementary Information:**

The online version contains supplementary material available at 10.1186/s13229-024-00618-0.

## Background

The core of the autism phenotype is centered around early developmental difficulties in the domains of social-communication (SC) and restricted repetitive behavior (RRB). Despite the core SC and RRB commonalities, autistic individuals markedly vary in the domain of motor development. It has been estimated that anywhere from 34 to 80% of autistic individuals show some form of motor impairment and/or delay [1–5]. Motor difficulties in autism are often associated with language delay [6–8], cognitive impairment [9–11], poorer developmental outcomes [12], and reduced life quality [13–15]. Because motor difficulties may affect a large percentage of autistic individuals, a recent debate has emerged regarding whether these difficulties should be added to the diagnostic criteria [1, 16–19]. However, the way in which motor abilities are affected in autism is quite heterogeneous. Motor difficulties range from delays in attaining early motor milestones to severe deficits in motor coordination that hinder daily living and adaptive functioning [14, 20]. Thus, a discussion regarding global motor impairment in autism is not sufficient and may not be helpful in the context of precision medicine and personalized intervention [21, 22]. A characterization of heterogeneous motor profiles in autistic individuals is needed and may help link to other latent profiles that extend into other key domains such as language, intellectual and adaptive functioning.

Understanding motor issues in autism may also be key to honing in on biological mechanisms that affect some autistic individuals. At a genetic level, it is known that individuals with highly penetrant but rare protein truncating de novo mutations associated with autism, also tend to show delays in the acquisition of early motor milestones (e.g., age at walking) [23–28]. Many of these rare highly penetrant mutations are known to converge on a final common pathway of dysregulated neurophysiological balance between excitatory and inhibitory (E:I) neuronal signaling in the brain [29, 30]. Synaptic E:I imbalance can attenuate signal-to-noise ratio in neural circuitry and enhance neural noise [31]. The higher level of noise in the brain could be reflected in the higher level of noise in motor circuitry. We hypothesize that a downstream consequence of higher neural noise specifically in the motor circuitry could lead to a behavioral prediction of enhanced motor noise—that is, increased variability when performing the same motor action repeatedly [32]. While variability is a ubiquitous and healthy feature of neuronal circuits [32–34], enhanced neural and motor noise has been proposed to be more pronounced on-average in autism [35–41].

**Fig. 1:**
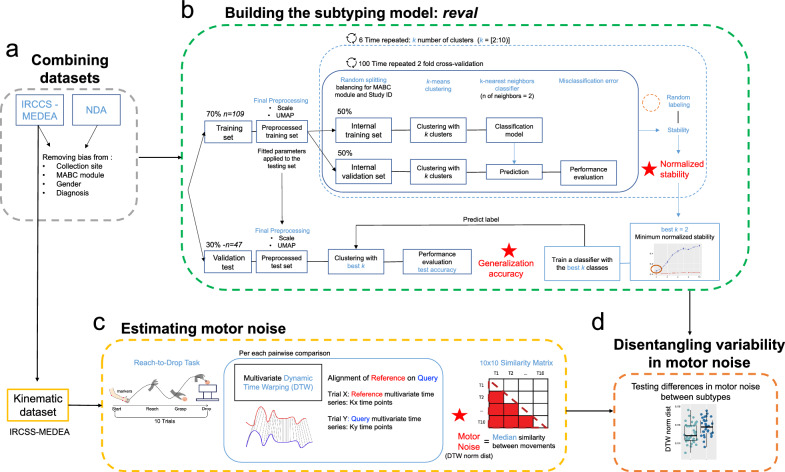
Overview of motor stratification and kinematic data analysis workflow. Panel **A** shows how IRCCS-MEDEA and NDA datasets are combined and how the initial preprocessing steps are implemented to remove confounding effects of originating study ID, MABC2 module, and sex. Panel **B** shows the workflow for stability-based relative clustering validation (*reval*) analyses that aimed to identify the optimal number of clusters (best k) that minimizes normalized stability in independent splits of the data (training and validation) and estimate the generalization accuracy of such optimal (best k) clustering solution. Panel **C** shows the analysis workflow for how we estimated motor noise from kinematics data acquired during a simple reach-to-drop task from the IRCCS-MEDEA dataset. Within this task, 10 repeat trials were administered and we used multivariate dynamic time warping (DTW) to align and compare motor kinematic trajectories across repeat trials. Motor noise is operationalized as the median similarity across trials (DTW dist norm) whereby higher estimates are indicative of more motor noise (i.e., increased dissimilarity between repeat trials). Panel **D** indicates the final step of hypothesis testing for subtype differences in motor noise

In this work we investigated the hypothesis of whether stratification of autism by clinical motor profiles leads to enhanced precision in parsing apart individuals that may be differentially affected by motor noise. First, we use an unbiased data-driven approach to identify whether there are discrete motor subtypes in autism, using clinical profiles of motor behavior assessed with a standardized test of motor ability—the Movement Assessment Battery for Children—2nd edition (MABC2). Second, we apply subtype labels to motor kinematics data from a simple reach-to-drop motor task (Fig. 1C) [42, 43] and test whether subtypes differ in terms of motor noise. Lastly, we divided the kinematic task into feedback and feedforward components [44] to assess whether motor noise is expressed differently by the subtypes in each segment.

## Methods

### NDA dataset

For the purposes of building a motor stratification model of autism, in March 2020 we downloaded all publicly available data from the National Institute of Mental Health Data Archive (NDA; https://nda.nih.gov) that included autistic (n = 62; n = 12 female), typically-developing (TD; n = 56; n = 12 female), or developmental coordination disorder (DCD; n = 23; n = 9 female) children aged 8–16 years old (mean age = 11.2; SD age = 1.73) whom also had a complete Movement Assessment Battery for Children—2nd Edition (MABC2) [45]. Available data originated from 3 different NDA collections: 2566 n = 38, 2254 n = 88, 2799 n = 15. A fourth data collection (collection ID = 2093) was identified yet excluded for the unusual data distribution (i.e., most of the individuals included had a score at the lower end of the MABC2 total score scale). NDA MABC2 data were then filtered to only include individuals between 3 and 16 years of age. Duplicate data were identified and dropped. Finally, if more than one MABC2 subscale domain was missing, the subject was dropped from the analysis. Global unique identifiers and collection IDs for all data utilized from NDA are reported in Supplementary Table 1.

### IRCCS-MEDEA dataset

In addition to data from NDA, we also compiled an in-house dataset which used the MABC2. The in-house dataset consisted of n = 94 (n = 17 female) autistic and n = 93 (n = 23 female) typically-developing (TD) children aged 3–12 years old (mean age = 7.34; SD age = 2.4), recruited at the Scientific Institute IRCCS Eugenio Medea (IRCCS-MEDEA) in Italy [46]. The autistic individuals had been recruited while undergoing either a clinical assessment (diagnostic or follow-up) or comprehensive rehabilitation program at the Child Psychopathology Unit of IRCCS-MEDEA, where all the testing occurred. The experimental procedure included the administration of the MABC2 [45] and the performance of 10 trials of a reach-to-drop motor task [42, 43, 46] while kinematic data were recorded with an optoelectronic system. Moreover, additional information was retrieved from each autistic individual's closest clinical record. They included information about: early development (i.e. the age at independent walk and the age of the first words), intellectual abilities (tested with the Griffith Mental Developmental Scales—3rd Edition (GMDS-3) [47], the Wechsler Preschool and Primary Scale of Intelligence—III (WPPSI-III) [48], the Wechsler Intelligence Scale for Children (WISC) 3rd and 4th edition [49, 50]) and autism core symptoms severity (using the Calibrated Severity Score (CSS), for the Total, the Social Affect (SA) and the Restricted and Repetitive Behavior (RRB) domains of the Autism Diagnostic Observation Scales- 2nd edition (ADOS-2) [51] and the Social Responsiveness Scales—SRS [52]). Supplementary Table 2A provides a description of the sample size for each of the investigated features.

To evaluate our second aim of whether motor noise was enhanced specifically within an autism motor subtype, we utilized kinematics data recorded using an optoelectronic system while children performed 10 repetitions of a simple upper-limb reach-to-drop motor task [42, 43]. Twenty-four autistic individuals and 14 typically developing did not complete all the 10 trials of the task and thus were excluded from the analysis.

### Movement Assessment Battery for Children—2nd edition (MABC2)

To assess motor profiles in young children we used the MABC2 [45]. MABC2 is a gold standard clinical test for the diagnosis of DCD in children aged 3 to 16 years old. However, it can be useful in assessing motor proficiency in a variety of developmental conditions, and it has been extensively used in the autistic population [3, 46, 53]. The MABC2 is composed of 3 subscales investigating specific aspects of motor coordination: the Manual Dexterity (MD) subscale, which refers to fine-motor coordination tasks; the Aiming and Catching (AC) subscale that includes oculo-motor activities; and the Balance (BL) subscale that tests both static and dynamic balance abilities. To cover a wide age range, the MABC2 is divided into 3 age bands (referred to hereafter as modules - module 1: 3–6 years; module 2: 7–10 years, module 3: 11–16 years) that assess the very same skills using age-appropriate tasks and activities. The advantages of using the MABC2 in autism is that the task’s instructions include a practical demonstration that can be followed by non-verbal autistic individuals. Furthermore, MABC2 is known to have high internal consistency (0.90) and excellent test–retest reliability (intraclass correlation coefficient = 0.97) in a DCD sample [54].

### Kinematic task—data acquisition and preprocessing

Motor kinematic data was collected during a simple reach-to-drop task previously described by Forti et al. [43] and Crippa et al. [42]. Kinematic data were collected at IRCSS-MEDEA. Each trial started with the child’s hand resting in a set position at a distance equal to 80% of each child’s forearm length from the ball support. The child was asked to complete 2 subsequential movements: (1) grasp a rubber ball (6-cm diameter), placed over a small support, and (2) drop it in a plastic “castle” with a 7 cm diameter hole on the top. The “castle” was a see-through square box (21 cm high and 20 cm wide) large enough not to require fine movements while dropping the ball. Task instructions were provided by a practical demonstration with no verbal cues so that non-verbal and minimally verbal autistic individuals could take part in the experiment. The same experimenter was present for all sessions in order to avoid any possible confounds due to the practical demonstration. Practice trials, the number of which varied according to each individual, were given to participants before recording in order to verify the child’s understanding of the task. The participants were allowed to interrupt the experiment at will in order to rest.

Each movement was recorded using an optoelectronic system (the SMART D from BTS Bioengineering—Garbagnate Milanese, Italy). Three-dimensional kinematic data was collected by eight infrared-motion analysis cameras at 60 Hz (spatial accuracy: 0.2 mm), located four per side at 2.5 m away from the participants. Passive markers (1 cm) were attached to the elbow, ulnar and radial surfaces of the participants’ wrists and to the hand dorsum on the fourth and fifth metacarpals (Fig. 1C). This resulted in a total of four body parts for kinematic trajectories to be recorded from. Subsequently, a dedicated software system (Smart Tracker, BTS Bioengineering—Garbagnate Milanese, Italy) was used to track and reconstruct the acquired movement by naming each single moving point recorded by the cameras in each time-frame. This allowed for frame-wise definition of a movement trajectory in 3-dimensional space coordinates (x, y, z). Data were then preprocessed in MATLAB (Mathworks—Natick, MA, USA) using a fifth-order Butterworth (8-Hz) low-pass filter. Although the task was presented to all children, 24 autistic and 14 TD children did not complete all 10 trials of the task, or chose to change hand (e.g., from left to right) during the task and were removed from the analysis. The final analysis was undertaken on n = 70 autistic and n = 79 TD individuals.

### Dataset combination and batch correction

For our first aim to stratify autism by MABC2 profiles, we first combined the IRCCS-MEDEA and NDA datasets to get the largest possible sample size for the stratification analysis. The final sample size was n = 328 individuals, aged 3 to 16 years old, split into n = 156 autistic (n = 29 females), n = 23 DCD (n = 9 females) and n = 149 TD (n = 35 females) children (Supplementary Table 2A–B). Because data originated from multiple sites (e.g., IRCCS-MEDEA and 3 other NDA datasets), we first implemented a batch correction technique to control for the variance attributed to factors that might introduce some systematic biases in the following analysis, such the originating study ID, the MABC2 module used and sex of participant (Fig. 1A). This batch correction was implemented as a linear model with each MABC2 subscale or total standardized score as the dependent variable and the originating study ID, MABC2 module, sex, and diagnosis as independent variables. Beta coefficients for originating study ID, MABC2 module and sex were used to project out variance related to those features before any further downstream statistical analysis. For the subsequent stratification model analysis, only the autistic subjects were utilized. For full reproducible analyses of all results presented in this work, please see https://github.com/IIT-LAND/motor_stratification_paper.

### Stability-based relative clustering validation analysis (*reval*)

To identify robust, stable, and reproducible subtypes based on motor profiles from the MABC2, we used a stability-based relative clustering validation approach called *reval* (https://github.com/IIT-LAND/reval_clustering) [55]. Practically, *reval* takes as input independent training and validation sets and selects the optimal number of clusters by looping across a range of possible clusters solutions (*k* = range[2:10]). For each clustering solution *reval* applies a repeated *n* cross-validation scheme that partions the training set repeatedly into the internal-training and internal-testing sets and then implements a clustering algorithm on each. A classifier is then fit to the internal-training set and is used to predict the clusters labels of the internal-testing set, which then allows for obtaining a performance metric - the misclassification error. *reval* uses the average of the misclassification error obtained across the *n* cross-validation repetitions for each of the possible *k* to select the optimal *k,* defined as the one having the lowest normalized *stability* (i.e., a measure that combines the classifier’s misclassification error with the misclassification after random labeling). The general idea is that the optimal cluster solution in terms of the number of clusters (*k*) is the most reproducible, hence a classifier trained on a first independent set (interval-training set) should be able to accurately classify the observation of another independent set (internal- testing set) resulting in higher classification accuracy and a lower misclassification error. Once the optimal *k* has been identified, *reval* applies that *k* as the number of clusters to identify in the held-out test set. It then trains a classifier on the original training set and uses it to predict the held-out test set labels. This classifier accuracy is called generalization accuracy and it is a performance metric that allows for interpreting if the clustering solution is thoroughly reproducible in independent datasets. For more details about *reval* please refers to our prior published work [55].

With respect to this work, preprocessed MABC2 data from autistic individuals was used as input for the *reval* analysis. A total of n = 156 autistic individuals were first split into *train* and *validation* sets using a 70–30 split scheme, while also balancing for originating study ID, sex, and MABC2 module. Before being entered as input features for *reval*, the three MABC2 subscales were scaled to a mean of 0 and a standard deviation of 1 (*sklearn.preprocessing.StandardScaler*) and then transformed using Uniform Manifold Approximation and Projection (UMAP) (n_neighbors = 30, min_dist = 0.0, n_components = 2, random_state = 42, metric = Euclidean). Those steps were done by fitting the models on the *train* set and applying them to both *train* and *validation* sets. Clustering and classification models were fit using k-means clustering and k-nearest neighbor classifier algorithms from the python *scikit-learn* library. To identify the optimal number of clusters via minimizing normalized stability, we used a twofold cross-validation scheme on the *train* dataset and searched through cluster solutions 2 through 10. The cross-validation scheme was repeated 100 times to ensure robustness. The identified optimal number of clusters was then used for clustering on the *validation* set. A classifier was then trained on the *train* set and utilized to predict the labels on the *validation* set to test the reproducibility of the cluster solution in a held-out sample. The accuracy of the classifier on the *validation* set is called *generalization accuracy* and describes how well the classification model fit to the *train* dataset can identify similar labels in the independent *validation* set (Fig. 1B). While stability-based relative clustering validation in *reval* tells us about the stability of clustering solutions, it does not test whether the actual solution is indicative of true clusters. Therefore, we followed up on the *reval* analysis by using the *sigclust* library in R to test whether the observed clustering solution significantly differs from the null hypothesis that the data originates from a single multivariate Gaussian distribution [56].

### Testing subtypes for differences in non-motor domains

Autism motor subtypes were examined for a number of phenotypic differences such as autism symptom severity, autistic traits, intelligence, and age at acquisition of developmental milestones such as walking and first words. For this analysis, only individuals from the IRCCS-MEDEA dataset were analyzed, since this was the only dataset that had presence of variables measuring these features. To measure intelligence, we utilized combined standardized scores (mean 100, SD = 15) across measures such as the GMDS-3 [57], WPPSI-III, and WISC 3rd and 4th edition [49, 50]. Autism symptom severity was measured with ADOS-2 SA and RRB calibrated severity scores (CSS) [51]. Autistic traits were measured with the SRS [52]. Age at achievement of early developmental milestones (i.e., age at independent walking, age at first words) were retrieved from clinical history data collected by clinicians during initial examination. Those measures were available for a portion of the entire IRCCS-MEDEA sample, hence the sample size for each hypothesis test changes accordingly (see Supplementary Table 2A). Hypothesis tests were conducted via Welch two-sample t-tests or Wilcoxon signed-rank tests when data significantly deviated from a Gaussian distribution (Supplementary Table 3A).

### Motor kinematic analyses

Motor noise was assessed via analysis of kinematic data from a simple reach-to-drop task from the IRCCS-MEDEA dataset. Here we defined motor noise as the degree of variability in movement trajectories between repeated trials on this task [32, 58]. To estimate variability between the 10 repeat trials of the task, we utilized multivariate dynamic time warping (DTW) implemented with the *dtw* function in the *dtw* library in *R* (distance metric: Euclidean, step pattern: Symmetric2, begin and end: close). For each individual, DTW resulted in a 10 × 10 similarity matrix. We defined motor noise as the median distance between trials, computed as the median in this 10 × 10 DTW similarity matrix. Larger values on this measure indicate higher levels of motor noise due to higher dissimilarity in movement trajectories between the 10 repeated trials (Fig. 1C). Given that the movements were already segmented so that data for each trial started and ended with the starting and ending of the movement, the DTW algorithm was forced to match the first and the last timeframe while comparing trials. Moreover, to avoid any bias given by the velocity in performing the movement, the *normalized distance* output was used for the subsequent analysis which represents the difference in the trajectories normalized by the total duration of the movement. Hypothesis tests for group differences in motor noise (e.g., DTW normalized distance) were examined with ANOVA and post hoc Welch two-sample t-tests.

### Examining motor noise during feedforward and feedback phases of reaching

It is well known in the literature that reaching actions can be characterized by two phases [44, 59–64]: (1) a first feedforward phase defined by the first deceleration peak after the max peak velocity, and (2) a subsequent feedback phase preceding the grasping of the object (Fig. 4A). These phases are thought to be underpinned by distinct neurocomputational mechanisms and thus are important to separate and examine for differences between autism motor subtypes. Therefore, in addition to examining motor noise for the entire reach-to-drop action, we also examined motor noise for these specific phases of the reach action. All of the same methods used to estimate motor noise with multivariate dynamic time warping were used in these analyses. The primary difference is that the movement trajectories were segmented into feedforward and feedback phases via identifying the first deceleration peak (DP) after the max velocity peak for the reaching action. The feedforward segment starts from the beginning of the reaching phase of the task up until the first deceleration peak, while the feedback phase starts with the first deceleration peak and ends with the reaching (Fig. 4A). Because the two phases of the reach action can be thought of as a within-subject factor, we modeled between-group differences as potential group*phase interactions within a linear mixed effect model that treated group and phase as fixed effects and modeled random intercepts grouped by subject ID. For n = 6 autistic individuals and n = 6 typically developing children we could not identify the 1st peak of deceleration after the 1st peak of velocity, hence the segmentation was not possible and they were dropped for this analysis step.

## Results

### Identification of autism motor subtypes with data-driven clustering

**Fig. 2:**
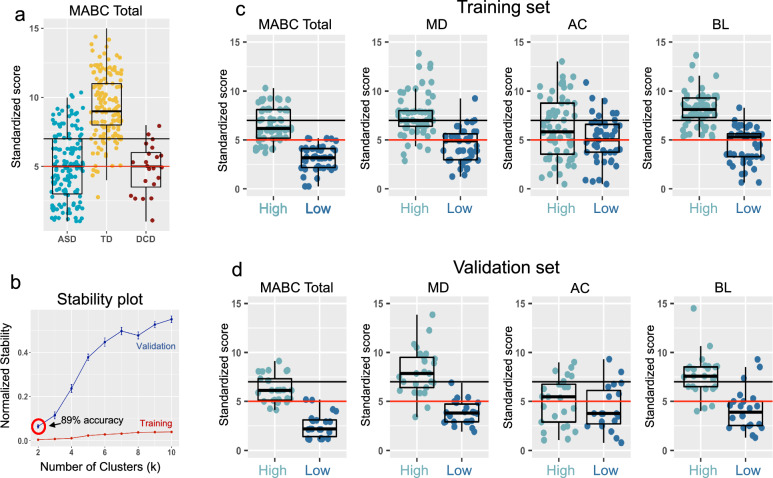
Autism motor subtypes. Panel A plots the MABC2 total standardized score for typically-developing (TD; yellow-orange), autistic (blue), and Developmental Coordination Disorder (DCD; maroon) individuals. The horizontal solid black and red lines represent cutoffs for ‘at risk of having a motor impairment’ (solid black line) and ‘have a motor impairment’ (solid red line) according to the MABC2 manual. With stability-based relative clustering validation (*reval*) analyses, the optimal clustering solution identified was k = 2, indicating two subtypes that could be identified with 89% accuracy in independent data. The two subtypes (Autism High, light blue; Autism Low, dark blue) are described with respect to total MABC2 score, and the MD, AC, and BL subscales of the MABC2 in the Training (C) and Validation (D) sets

The primary goal of this work was to examine how motor behavior in autism may vary and to test whether there are distinct autism motor subtypes. Notably, the variability in motor performance within the autistic population is substantial, ranging from severely impaired to within the normative range (Fig. 2A). Before digging into analyses that explore this heterogeneity, it is crucial to first describe how autism can be characterized through a case–control comparison involving both a typically developing (TD) group and a non-autistic comparison group with specific motor impairments, such as Developmental Coordination Disorder (DCD). To achieve this goal, we first compiled publicly available data from NDA with our own in-house dataset (IRCCS-MEDEA) that utilize MABC2 (autism n = 156, DCD n = 23, TD n = 93; Fig. 1A; Supplementary Table 2A–B). Large group differences are apparent in MABC2 total standardized score (*F* = 148, *p* = 2.01e−46), which can be described as lower scores in autism compared to TD (*t* = − 16.37, *p* = 1.42e−43, *Cohen’s d* = 1.87), but no case–control differences when autism is compared to DCD (*t* = 0.29, *p* = 0.77, *Cohen’s d* = 0.05) (Fig. 2A).

The case–control analyses may indicate that very prominent motor impairments are a key characteristic of autism as a whole. However, before making this interpretation, it is important to analyze whether the autism group could be split into robust, stable, and reproducible subtypes [65, 66]. Evidence supporting the notion that autism can be split into subtypes may help refine the precision of our interpretations about motor skills for specific types of autistic individuals. To test this, we analyzed multivariate clinical motor profiles from the MABC2 for evidence of robust, stable, and reproducible autism subtypes with stability-based relative clustering validation (*reval*) analysis [55, 67] (Fig. 1B). Our analysis revealed the existence of two autism motor subtypes with high generalization accuracy (89%) in independent data (Fig. 2B). Evidence of cluster separation can be discerned by comparing the data to simulated data originating from a single multivariate Gaussian null distribution. This analysis shows robust evidence of separated clusters that highly deviated from the single multivariate Gaussian null distribution (SigClust *p* = 9.999e−05) [56]. The subtypes can be described as relatively ‘High’ (56%, N = 87) versus ‘Low’ (44%, N = 69) levels of motor proficiency, with subscales such as MD (*Cohen’s d* for the training set = 1.55, validation set = 2.18) and BL (*Cohen’s d* for the training set = 2.06, validation set = 1.73) showing the largest differentiation between the subtypes (Fig. 2C–D), whereas much less differentiation exists between subtypes in the AC subscale (*Cohen’s d* for the training set = 0.43, validation set = 0.54). Notably, the subtypes are identified with high generalization accuracy without visible evidence of hard cutoffs (Fig. 2C–D). However, it is apparent that when data is plotted with traditional MABC2 cutoffs for motor impairment, the relatively ‘Low’ subtype scores at or below this threshold while the relatively ‘High’ subtype is largely above this cutoff for a majority of individuals (Fig. 2C–D).

With robust and stable subtype labels known, we can re-evaluate our model of group differences considering the autism subtypes along with TD and DCD. Using the Akaike Information Criterion (AIC) statistic [68], the subtype model better explains variance in the total MABC2 standardized scores (ΔAIC = 137). We can also describe how the ‘Low’ and ‘High’ subtypes compare relative to the TD and DCD comparison groups. The ‘Low’ subtype is more than 3 standard deviations below the TD group (*t* = − 26.98, *p* = 4.43e−70, *Cohen’s d* = 3.22) and is also more impaired than the DCD group, even though the effect size is much less pronounced (*t* = − 2.99, *p* = 0.005, *Cohen’s d* = 0.89). In contrast, the ‘High’ subtype still shows lower on-average scores compared to TD (*t* = − 11.81, *p* = 2.19e−25, *Cohen’s d* = 1.46), but score higher than DCD (*t* = 4.94, *p* = 2.69e−5, *Cohen’s d* = 1.3). This indicates that the overall lack of case–control difference between autism and DCD was driven primarily by the ‘Low’ autism subtype.

**Fig. 3:**
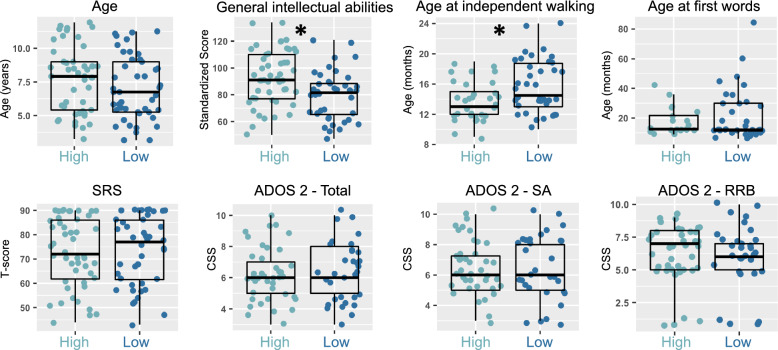
Characterization of autism motor subtypes by age, general intellectual level, early developmental milestones, and autistic traits and symptom severity. In this figure we describe subtypes (Autism High, light blue; Autism Low, dark blue) in terms of age, general intellectual level, age at acquisition of early developmental milestones (age at independent walking and first words), and autistic trait and symptom severity as measured by the SRS and ADOS-2 respectively. Asterisks indicates *p* < 0.05

We next tested for subtype differences on skills outside of the motor domain. Data for this follow-up analysis were available only for the IRCCS-MEDEA dataset. Subtypes did not significantly differ by age (*Wilcoxon Z* = 1270, *p* = 0.2). Regarding other general intellectual ability and achievement of early developmental milestones, we discovered that the relatively ‘Low’ motor skill autism subtype shows significantly lower scores in general intellectual ability (*t* = 3.3, *p* = 0.001, *Cohen’s d* = 0.7) and higher age at independent walking (*Wilcoxon Z* = 417, *p* = 0.015, *Cohen’s d* = 0.67). However, no significant difference was apparent for age at first words (*Wilcoxon Z* = 343.5, *p* = 0.95). Similarly, no significant differences were apparent for ADOS-2 CSS scores [69] or the SRS [52] (Fig. 3; Supplementary Table 3A).

### Autism motor subtypes are differentiated by motor noise

**Fig. 4:**
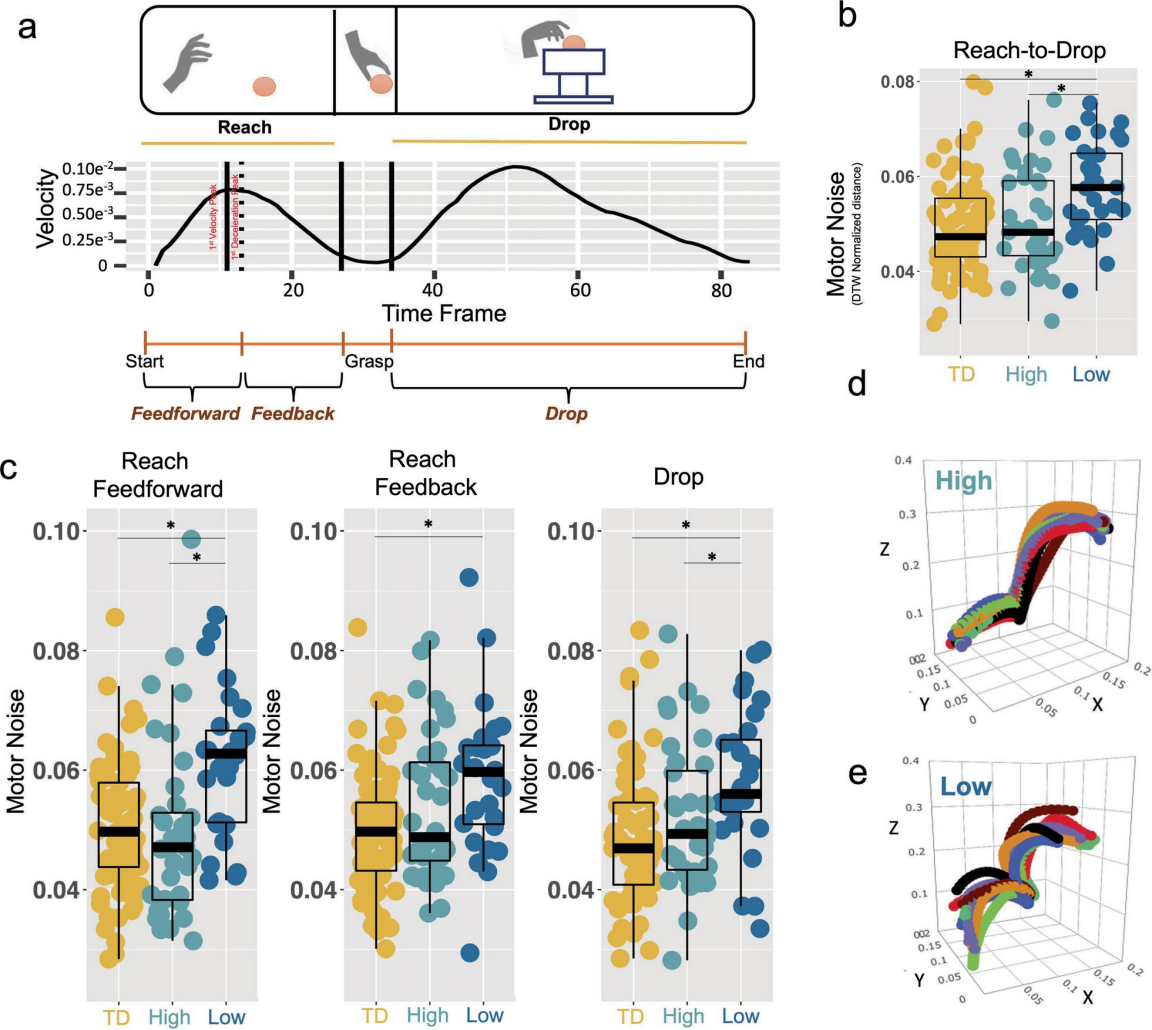
Enhanced motor noise specific to the poor motor skill autism subtype. Panel **A** shows the reach-to-drop task, segmented into reach and drop actions, and with the reach action split into feedforward and feedback phases according to the deceleration peak. The velocity plot represents the instantaneous velocity of the medial wrist marker of a random participant. Panel **B** shows group differences (TD, yellow-orange; Autism High, light blue; Autism Low, dark blue) when motor noise is measured across the entire reach and drop actions. Panel **C** shows group differences when the task is split into reach and drop actions and with the reach action split into feedforward and feedback phases. Panels D and E show the variability across trials for a randomly selected participant. Trajectories for one body part (lateral wrist) are displayed in their native 3D space (x, y, z). Panel **D** displays one example subject from the 'High' subtype, whereas Panel E displays an example subject from the 'Low subtype'. Each colored line indicates one trial. Asterisks indicate *p *< 0.05

Thus far, we have demonstrated that autism can be separated into at least two subtypes by clinical motor profiles. The second aim of this work was to test whether such subtypes are highly differentiated in terms of motor noise. To estimate motor noise we analyzed motor kinematics acquired during repetitions of a simple reach-to-drop task (Fig. 1C) [42, 43, 46] from the IRCCS-MEDEA dataset (n = 149, autism High n = 37, autism Low n = 33, TD n = 79). We operationalized motor noise for each subject as the degree of variability between movement trajectories during 10 repeat executions of the task [32]. We used multivariate DTW to estimate the variability. DTW output measures the distance between trial trajectories. Higher DTW (normalized) distance between trajectories indicates more variability and, as a result, higher levels of motor noise. For each individual, we apply DTW in a pair-wise fashion across the 10 trials resulting in a 10 × 10 similarity matrix. We defined motor noise as the median distance between trials, computed as the median in this 10 × 10 DTW similarity matrix. We find highly significant differences between groups in motor noise (*F* = 8.7, *p* = 2.5e−4), driven by enhanced motor noise in the relatively ‘Low’ subtype compared to the relatively ‘High’ subtype (*t* = − 3.2, *p* = 0.002, *Cohen’s d* = 0.77) and compared to TD children (*t* = − 4.1, *p* = 1.33e−4, *Cohen’s d* = 0.85). In contrast, there was no difference between the relatively ‘High’ subtype and TD children (*t* = − 0.4, *p* = 0.66, *Cohen’s d* = 0.08) (Fig. 4B). This result reflects poorer precision, and thus higher variability, in repeat executions of the action that is specific to the relatively ‘Low’ subtype. Illustrative examples of this effect can be seen in Fig. 4D, E. It is visually evident that the individual in the ‘High’ subtype shows very similar trajectories for each of the 10 repeated executions. In contrast, the example individual in the ‘Low’ subtype shows much more variable trajectories for each execution.

### Enhanced motor noise during the feedforward phase of reaching

The reaching component of our task can be broken down into two functionally separable components—feedforward and feedback phases. The transition between these phases is demarcated by the end-point (i.e., wrist) transport deceleration peak [63]. Therefore, we re-examined motor noise when the data is split into these two phases. Linear mixed effect modeling was able to identify a significant group*phase interaction (*F* = 3.5, *p* = 0.03; Supplementary Table 4) which is indicative of group differences in motor noise that are dependent on the phase (feedforward or feedback). Follow-up tests showed that the autism subtypes are highly differentiated during the feedforward phase (*t* = − 3.56, *p* = 7e−4, *Cohen’s d* = 0.87), but were not different during the feedback phase (*t* = − 1.55, *p* = 0.12, *Cohen’s d* = 0.39) (Fig. 4C). Examination of motor noise for the drop action also indicated that the ‘Low’ subtype showed more motor noise than the ‘High’ subtype (*t* = − 2.05, *p* = 0.045, *Cohen’s d* = 0.51) (Fig. 4C). Comparing the autism subtypes to TD children, we observed similar levels motor noise in the relatively ‘High’ subtypes in both feedforward and feedback phase and in the drop action. In contrast, motor noise of the relatively ‘Low’ subtype was always higher with respect to TD children (Supplementary Table 5). Overall, these results demonstrate that enhanced motor noise is a specific characteristic of the relatively ‘Low’ motor skill autism subtype and that this effect could be most pronounced within neural circuitry that supports computations critical for feedforward processing.

## Discussion

In this work we aimed to study heterogeneity in motor ability in autism. Past work has indicated that motor issues are a very prominent feature of autism and that it could be potentially important to consider adding this domain to the diagnostic criteria in the future [1, 17]. Congruent with these ideas, if one were to simply use cut-off scores on the MABC2, we would find that a large majority of autistic individuals in our sample (74%) show medium to severe motor impairments, while only 26% possess motor skills in line with age-expected norms. However, this way of analyzing the data does not rigorously test whether autism is indeed a single group or a collection of different motor subtypes. Our work shows first and foremost that when considering motor ability in autism, the data do not conform to a single unitary group. Rather, autism can be split in an unbiased and data-driven manner into two subtypes—relatively ‘High’ versus ‘Low’ groups. These ‘High’ versus ‘Low’ subtype labels are intended as descriptive terms referencing the scores of MABC2 test and are not meant to be interpreted in relation to functioning level of each subtype. While the autism group shows on-average lower standardized scores on the MABC2, this lower level of motor ability is clearly driven by the ‘Low’ subtype, which considerably drives down the overall average score of the autism group. However, even the relatively ‘High’ subtype identified here is still on-average lower than the TD group (*Cohen’s d* = 1.46). Nevertheless, this relatively ‘High’ group is still higher than a non-autistic group of individuals with very pronounced motor impairments (e.g., the DCD group; *Cohen’s d* = 1.3). Finally, the percentages of individuals in these two subtypes (Low = 44%; High = 55%) do not easily conform to the percentages seen when one uses standardized cutoff scores on the MABC2 (e.g., 74% vs. 26%). This result illustrates the need to characterize autistic individuals not only by where they stand relative to TD norms, but also with regards to how they are grouped within the autism population [67].

After identifying autism motor subtypes, we next found that the relatively ‘Low’ autism motor subtype could be characterized by enhanced motor noise during a simple reach-to-drop task where fine-grained motor kinematics were measured. Motor noise is defined as the degree of variability in repeat motor actions [32] and is thought to be a downstream consequence of neural noise within motor circuitry [31]. The concept of neural noise can be linked to long-standing ideas in autism research, such as the E:I imbalance theory [29, 30]. It is known that many highly penetrant rare genetic mutations associated with autism also highly dysregulate E:I balance [29, 30] and these types of genetic mutations are often associated with delays in acquiring early motor milestones [23–28]. Enhanced motor noise specific to the relatively ‘Low’ autism motor subtype may be revealing of very different neurobiological mechanisms linked to synaptic E:I imbalance in motor circuitry. With regards to how these insights could help drive future work, we suggest that new studies could utilize our motor stratification model to examine how these motor subtypes might be different with respect to biomarkers relevant to E:I imbalance in neuroimaging data [70]. If E:I imbalance is a key neurobiological issue in the ‘Low’ motor subtype, it may be important to utilize our stratification model in clinical trials that target key E:I mechanisms [71–73]. Other future work could examine how rare variant or polygenic genomic architecture may affect motor circuitry in a differential manner in phenotypically-defined autism subtypes where motor skills are the central differentiating factor.

While motor noise highly differentiated the subtypes for trajectories analyzed across the entire reach-to-drop task, we also discovered that enhanced motor noise in the ‘Low’ subtype may be most pronounced for the feedforward phase of the initial reach action. This result is consistent with previous studies that provided evidence for alterations in the feedforward-based phase [35, 74, 75]. This result is also important with respect to the hypothesized different neurocomputational mechanisms that underlie feedforward versus feedback motor control. Motor control is based on the integration of feedforward action planning and feedback-based control processes. Feedforward processing derive from internal representations of the action that specify a relatively coarse motor output prior to its initiation, while feedback processes fine-tune the motor output on the fly, relying on sensory feedback and often applying corrective adjustments [76]. Action representations, as well as the neural machinery required to adapt them to incoming sensory information, are believed to rely on the cerebellum [77, 78]. Altered cerebellar function during development might play a key role in contributing to both motor and non-motor alterations in autism [79]. Altered feedforward and feedback mechanisms are also associated with the severity of communication impairments in autism and could potentially reflect the respective contributions of the anterior and posterior cerebellum [80]. Reduced motor noise during the feedforward phase for the ‘High’ autism motor subtype suggests that this subgroup, rather than having better feedback-based correction abilities, are characterized by relatively more preserved representation of actions.

In contrast to the sharp differences between autism motor subtypes in terms of general intellectual ability, acquisition of early motor milestones, and motor noise, these subtypes were not highly differentiated in terms of age, autistic traits, or autism symptom severity. This lack of differentiation in autistic traits and core autism symptom severity is important because it potentially underscores the orthogonal nature of motor versus core diagnostic features of autism (e.g., SC and RRB domains). An emerging literature is building indicating that the single diagnostic label of autism is not enough for understanding clinical and biologically important features within autistic individuals [66, 81]. Rather than looking to core SC and RRB features, it seems that a constellation of related features that do not represent the core features of autism—such as motor, language, intellectual, and adaptive functioning—may better separate out important clinical and biological distinctions within the autism population. Supporting this statement, there is evidence showing that motor difficulties in autism tend to highly co-occur with language delay [6–8], cognitive impairment [9–11], poorer developmental outcomes [12], and reduced life quality [13, 15]. While in this specific work we did not find a significance difference in terms of age at first words, other work reports that individuals with very poor early language outcome tend to also have extensive issues in motor, non-verbal cognitive ability, and adaptive functioning, and also have very different structural and functional neural mechanisms underpinning their difficulties [82–85]. A theoretical advance forward for the field would be to put together these findings under a model that supports the fact that a primary split in the autism population should be between individuals with very pronounced issues in this constellation of non-core features in motor, language, intellectual, and adaptive functioning. We have proposed such a theory and have provided initial evidence in support of this subtyping model [67]. The current work matches the predictions of our model and provides further empirical support for it. Furthermore, although this work cannot be directly translated into clinical practice, it may have some future clinical implications. The stratification of autism into motor-defined subtypes might help identify autistic individuals who require specialized intervention programs to improve their motor development. In this regard, we also suggest that our operationalization of motor noise might be a possible behavioral measure of motor intervention efficacy.

## Limitations

Several limitations and caveats must be noted for this work. First, while our goal was to examine the largest sample size available via combining all available MABC2 data from NDA and our in-house dataset (IRCCS-MEDEA), it may be that a larger sample size is needed to make stronger generalizations and cover the entire autism spectrum. Second, the MABC2 has been psychometrically evaluated with respect to DCD populations, but not in autism. A similar psychometric evaluation of MABC2 in the autism population is necessary for future work. Third, a more comprehensive assessment of motor noise could be achieved with kinematics data across a variety of different motor tasks (e.g., gait, jumping, pointing, clapping) to test the generalizability of the operationalization of motor noise as kinematic variability across repeated trials of the same task. Fourth, the analysis of differences in intellectual abilities between subtypes relies solely on composite IQ scores. However, due to the variability among IQ subscales, further investigation is required to elucidate the influence of each index and to examine potential differences between subtypes. Lastly, genetic and electrophysiological data was not available for the datasets analyzed in the current work. Thus, discussion about genomic mechanisms that potentially lead to E:I imbalance is speculation for future work to test.

## Conclusions

In conclusion, we have shown evidence that autism can be split into two subtypes based on clinical motor profiles measured by the MABC2. These subtypes show other differences in general intellectual ability, acquisition of early motor milestones, and motor noise. However, motor-defined subtypes are not different with regards to autistic symptomatology. Our findings fit with general findings that autism can be stratified into robust/stable and discrete subtypes. Such motor subtypes may be very relevant within the bigger picture of autism subtypes that share issues across language, intellectual, and adaptive functioning, but are likely orthogonal to issues within the core autism domains of SC and RRB.

### Supplementary Information

Additional file1.

## Data Availability

NDA data utilized in this work can be found in the National Institute of Mental Health Data Archive (NDA; https://nda.nih.gov). NDA global unique identifiers (GUID) and collection IDs are provided in Supplementary Table 1. IRCCS-MEDEA data can be made available upon reasonable request.

## Abbreviations

TD: Typically-developing
DCD: Developmental coordination disorder
MABC2: Movement Assessment Battery for Children 2nd edition
SC: Social-communication
RRB: Restricted repetitive behaviors
E:I: Excitation-inhibition
NDA: National Institute of Mental Health Data Archive
IRCCS-MEDEA: Scientific Institute IRCCS Eugenio Medea
GMDS-3: Griffith Mental Developmental Scales, 3rd edition
WPPSI-III: Wechsler Preschool and Primary Scale of Intelligence-III
WISC: Wechsler Intelligence Scales for Children
ADOS-2: Autism Diagnostic Observation Scales-2nd edition (ADOS-2)
CSS: ADOS-2 Calibrated Severity Score
SA: Social affect
SRS: Social Responsiveness Scale
MD: Manual dexterity subscale of the MABC2
AC: Aiming and catching subscale of the MABC2
BL: Balance subscale of the MABC2
*reval*: Stability-based relative clustering validation
UMAP: Uniform Manifold Approximation and Projection
DTW: Dynamic time warping
ANOVA: Analysis of variance
DP: Deceleration peak
GUID: Global unique identifier
AIC: Akaike information criterion

## Code availability

Analysis code to reproduce the analyses and figures is available on GitHub (https://github.com/IIT-LAND/motor_stratification_paper). The *reval* Python library can be found on GitHub (https://github.com/IIT-LAND/reval_clustering) and the documentation at https://reval.readthedocs.io.
